# Targeting Pediatric Glioblastomas by Combining OLIG2 Inhibitor CT-179 with Fractionated Radiation in a Panel of Patient-Derived Orthotopic Xenograft Mouse Models

**DOI:** 10.3390/ijms27031543

**Published:** 2026-02-04

**Authors:** Holly Lindsay, Yuchen Du, Lin Qi, Huiyuan Zhang, Sibo Zhao, Frank K. Braun, Mari Kogiso, Clifford Stephan, Gordon Alton, Gregory Stein, Graham Beaton, Santosh Kesari, Steve Neuhauser, Tim Stearns, Jeff Chuang, Emily L. Jocoy, Carol J. Bult, Beverly Teicher, Malcolm A. Smith, Xiao-Nan Li

**Affiliations:** 1 Texas Children’s Cancer Center, Texas Children’s Hospital, Baylor College of Medicine, Houston, TX 77030, USA; holly.lindsay@childrenscolorado.org (H.L.); yuchdu@luriechildrens.org (Y.D.); qilin23@mail.sysu.edu.cn (L.Q.); yongganhei2years@gmail.com (H.Z.); sibo.zhao@cookchildrens.org (S.Z.); fkbraun@outlook.com (F.K.B.); mari.kogiso@gmail.com (M.K.); 2 School of Medicine, University of Colorado Anschutz Medical Campus, Children’s Hospital Colorado Center for Cancer and Blood Disorders, Aurora, CO 80045, USA; 3 Ann & Robert H. Lurie Children’s Hospital of Chicago, Northwestern University Feinberg School of Medicine, Chicago, IL 60611, USA; 4 Guangxi Key Laboratory of Tumor Immunology and Microenvironmental Regulation, Guilin Medical University, Guilin 541004, China; 5 Ben Towne Center for Childhood Cancer and Blood Disorders, Seattle Children’s Research Institute, Seattle, WA 98101, USA; 6 Cook Children’s Neuro-Oncology, Dallas, TX 76104, USA; 7 Department of General, Visceral, and Transplant Surgery, Ludwig-Maximilians-University Munich, 80336 Munich, Germany; 8 Health Science Center, Texas A & M University, Houston, TX 77843, USA; cstephan@tamu.edu; 9 Curtana Pharmaceuticals, Inc., Austin, TX 78759, USA; altonyx44@gmail.com (G.A.); gregory.stein@curtanapharma.com (G.S.); gbepigen@gmail.com (G.B.); santoshkesari@gmail.com (S.K.); 10 Department of Translational Neurosciences, Pacific Neuroscience Institute, Saint John’s Cancer Institute at Providence Saint John’s Health Center, Santa Monica, CA 90404, USA; 11 PIVOT Coordinating Center, The Jackson Laboratory, Bar Harbor, ME 04609, USA; steven.neuhauser@jax.org (S.N.); tim.stearns@jax.org (T.S.); jeff.chuang@jax.org (J.C.); emily.jocoy@jax.org (E.L.J.); carol.bult@jax.org (C.J.B.); 12 National Cancer Institute, Bethesda, MD 20892, USA; beverly.teicher@nih.gov (B.T.); smithm@ctep.nci.nih.gov (M.A.S.); 13 Robert H. Lurie Comprehensive Cancer Center, Northwestern University Feinberg School of Medicine, Chicago, IL 60611, USA

**Keywords:** OLIG2, pediatric brain tumor, GBM, PDOX, radiation, therapeutic efficacy

## Abstract

The poor clinical outcomes of pediatric high-grade glioma (pHGG) highlight the urgent need for new therapies. Oligodendrocyte lineage transcription factor 2 (*OLIG2*) is a pro-mitotic transcription factor highly expressed in glioma stem cells and may represent a novel therapeutic target. To evaluate the therapeutic efficacy of an *OLIG2* inhibitor CT-179 in pHGG, we determined the *OLIG2* mRNA expression in 10 patient-derived orthotopic xenograft (PDOX) models. In vitro activities of CT-179 were analyzed in monolayer and neurosphere cells (0–10 µM) with and without radiation (XRT) (0–8 Gy), brain penetration was evaluated in tumor-bearing PDOX mice, and in vivo efficacy was determined at 15–240 mg/kg (oral) alone or combined with XRT (2 Gy/day × 5 days). Changes in animal survival times were analyzed using the Kaplan–Meier method, followed by pair-wise comparisons. Increased *OLIG2* mRNA expression was detected in seven out of ten PDOX models. CT-179 inhibited cell viability in a time- and dose-dependent manner in all eight pGBM xenograft tumors (IC_50_ 0.03–10 µM) and was potentiated by XRT (0.03–1 µM). Oral gavage (24 mg/kg) of CT-179 for 5 days led to effective penetration in mouse cerebrum (3232.7 ± 569.2 ng/g), cerebellum (1563.3 ± 269.6 ng/g), brain stem (1685.3 ± 309 ng/g), and PDOX tumors (1814 ± 110.3 ng/g) vs. 361.3 ± 1.5 ng/mL in serum. CT-179 alone was not active at 200 mg/kg in four models, although it was moderately effective at 240 mg/kg in one model. When combined with XRT, a significant extension of animal survival times was observed in two out of four models. Doses needed to eliminate OLIG2 expression in vitro varied from 0.3 to >1 µM in pGBM cells. In summary, our data showed that orally administered CT-179 penetrated the blood–brain barrier (BBB) and exhibited potential for inhibiting pGBM growth when combined with XRT.

## 1. Introduction

Pediatric high-grade glioma (pHGG), previously known as glioblastoma (GBM), remains one of the deadliest brain cancers in children younger than 21 [1,2]. Due to the developmental nature of childhood brains, therapy-related toxicity often leads to neurological deficits in pediatric patients, resulting in decreased quality of life. New therapeutic targets and strategies are therefore desperately needed.

Oligodendrocyte lineage transcription factor 2 (*OLIG2*) exhibits multiple features that make it an attractive target in GBMs. Located at chromosome 21q22, *OLIG2* is expressed almost exclusively in the central nervous system (CNS). It codes for a transcription factor that regulates fetal and adult oligodendrocyte, glial cell, and motor neuron development and promotes cellular differentiation from neural progenitors in the subventricular zone and spinal cord [3,4,5,6,7,8,9,10,11,12,13,14,15,16,17,18,19,20,21,22]. Over-expression of *OLIG2* has been documented in various human cancers, including leukemia, melanoma, lung cancer, and breast cancer [5]. Importantly, *OLIG2* is also highly expressed in human gliomas, including astrocytomas, oligodendrogliomas, oligoastrocytomas, and diffuse intrinsic pontine gliomas (DIPGs), as well as in 40–77% of medulloblastomas [4,7,10,11,15,16,17,20,21,22,23,24,25,26]. Specifically in pediatric brain tumors, expression of OLIG2 is highest in astrocytic tumors and low in ependymoma; notably, tumors arising in the supratentorial deep midline structures have higher OLIG2 expression than tumors from other anatomic locations [11]. Additionally, expression of OLIG2 in cerebellar neuronal progenitor cells can induce the formation of medulloblastoma [17], while *OLIG2*-null neural progenitors are more susceptible to radiation-induced and genotoxic damage [6]. Targeting OLIG2 has recently been shown to suppress recurrence [27] and alter the targetable stem cell fate [28] of SHH medulloblastoma.

*OLIG2* expression is closely associated with rapid cell proliferation, invasion, and stem cell populations. It is expressed in the majority of rapidly dividing glioma progenitor cells, characterized by Ki67 expression, and glioma stem cells, characterized by CD133 expression, in addition to normal-cycling neural stem cells [3,4,8,11,13,26,29,30,31]. *OLIG2* exerts its proliferation functions when phosphorylated at a triple serine motif in the amino-terminal domain; when phosphorylated, the protein is pro-mitotic and prevents cell cycle exit in both normal and malignant neural progenitor cells [3,23]. Expression of OLIG2 is induced by sonic hedgehog signaling and promoted by fibroblast growth factor receptor signaling [21]. The transcription factor is additionally expressed in the proliferating parenchymal cells of the developing human pons and again in the ventral pons during the middle of the first decade of life [30]. A subset of cells from patient-derived DIPG neurosphere culture strongly expresses *OLIG2* [25,30]. *OLIG2* has been identified as one of four core transcription factors that can de-differentiate GBM cells into stem cells with the ability to form tumors in vivo [26,32]. In genetically engineered mouse models of adult anaplastic astrocytoma and oligodendroglioma, tumor growth was shown to require functional *OLIG2* [4,8,9,11,33]. Additionally, *OLIG2* expression is higher in invasive glioma cells than in tumor core cells, with *OLIG2* inhibition leading to decreased invasiveness and downregulation of neural stem markers [34,35].

Molecularly, OLIG2 functions in direct opposition to *TP53*. In both neural progenitor cells and high-grade glioma cells, OLIG2 over-expression suppresses *TP53*-mediated responses to antineoplastic therapies by decreasing the post-translational acetylation of *TP53.* In contrast, cells with decreased OLIG2 levels undergo apoptosis and growth arrest when exposed to chemotherapeutics and XRT, even in the setting of low *TP53* function [3,6,21,23,24,26,31]. Additionally, OLIG2 negatively regulates the expression of p21, a cell cycle protein that inhibits the proliferation of stem cells and functions as a tumor suppressor, leading to increased expression of tumorigenic Cyclin D and CDK4/6 [21,24,31].

In this study, we utilize a panel of 10 PDOX mouse models to examine the expression levels of OLIG2, determine the anti-tumor activities of a new *OLIG2* inhibitor (CT-179) in vitro in monolayer and neurosphere cultures and in vivo in 4 PDOX models when administered alone and in combination with radiation, and quantify its penetration into PDOX tumors and mouse brains compared with blood concentrations. Our goal is to evaluate the potential of targeting OLIG2 with CT-179 in pediatric brain tumors.

## 2. Results

### 2.1. OLIG2 Is Over-Expressed in Pediatric GBM Cells

A total of 10 PDOX models of pediatric malignant gliomas were included (Table 1). Among them, four models were derived from treatment-naïve tumors and six from recurrent tumors, each with distinct sets of mutated genes.

**Figure 1:**
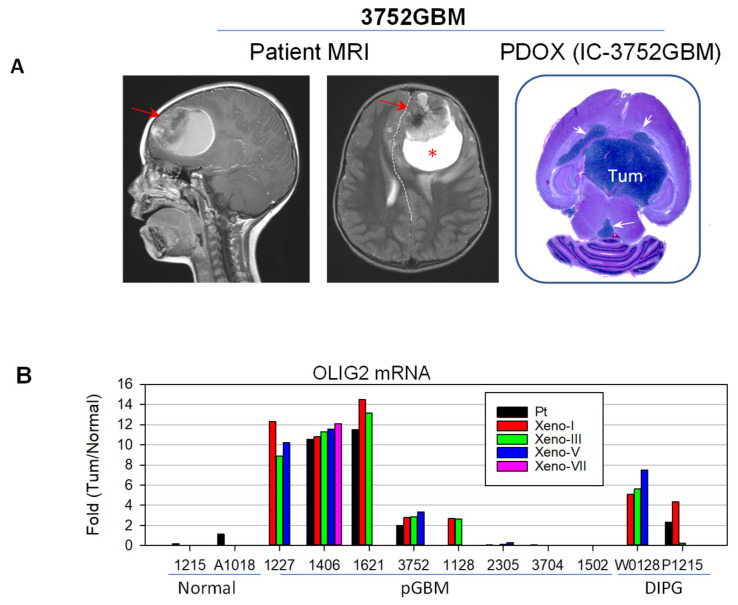
PDOX models of pediatric GBM. (**A**) Representative MRI and whole mouse brain section from pGBM 3752, from which the animal model IC-3752GBM was derived. Note the large frontal tumor (arrow) with massive edema (*), which displays remarkable space-occupying effects, the midline shift (dotted line) in the patient tumor, and a massive intra-cerebral xenograft (Tum) that spread to CSF (arrows). (**B**) Over-expression of OLIG12 mRNA in PDOX models of pGBMs compared with normal brain tissues. Data were extracted from gene expression profiling.

The expression of *OLIG2* mRNA in pGBM patient tumors and PDOX mouse models (Figure 1A) over serial sub-transplantations (from passage I up to VII) was derived from gene expression profiling with Illumina arrays and compared with two childhood normal cerebral tissues obtained from autopsy (Figure 1B). As shown in Figure 1, high-level (>10-fold) OLIG2 mRNA expression was detected in three out of eight pGBM tumors, and medium-level (2~10-fold) expression was detected in two out of eight pGBM tumors and two out of two DIPG tumors. In four models (three pGBM and one DIPG) in which original patient tumors were available, the increased *OLIG2* mRNA observed in the patient tumors was well maintained in the PDOX tumors during serial sub-transplantations, suggesting that *OLIG2* over-expression is frequent in pGBM and DIPGs. In two out of eight pGBM models, the very low levels of OLIG2 mRNA expression also suggest that OLIG2 may not be an important driver of tumor growth, which, in turn, highlights the inter-tumoral heterogeneity of pGBMs.

### 2.2. CT-179 Suppressed pGBM Proliferation In Vitro

**Figure 2:**
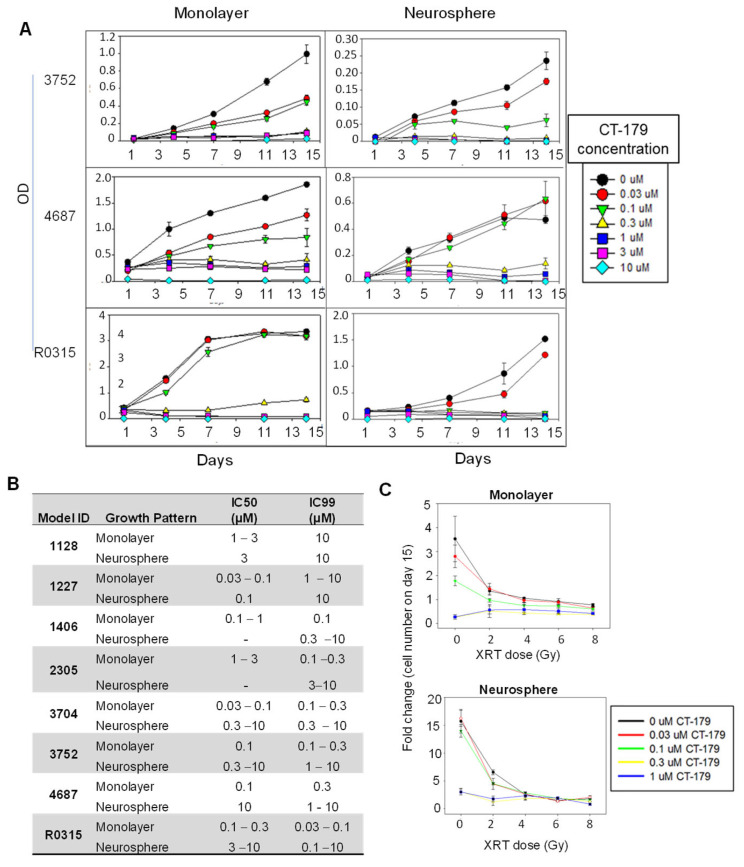
In vitro anti-tumor activities of CT-179. (**A**) Representative graphs showing the time- and dose-dependent inhibition of tumor cell proliferation. (**B**) Summary IC50 and IC99 of CT-179 in monolayer and neurosphere cells during the 14-day in vitro treatment. (**C**) Fold changes of cell number on day 15 in cells treated with CT-179 (0.03–1 µM) in combination with radiation (XRT) (2–8 Gys).

Since the cellular populations between monolayer and neurospheres of gliomas were different, and our previous studies showed that effective suppression of both monolayer and neurosphere cells predicted in vivo treatment success [36], we examined the anti-tumor activities of CT-179 in matching pairs of monolayer and neurosphere cultures from eight pGBM models for which in vitro growth was successful. These models expressed different levels of OLIG2, i.e., high (>10-fold, *n* = 2), medium (>2-fold, *n* = 2), and low (<2-fold, *n* = 2), and were treated with six different concentrations of the OLIG2 inhibitor CT-179, ranging from 0.03 µM to 10 µM, for 13–14 days. Dose- and time-dependent inhibition of cell proliferation was observed (Figure 2A). When neurospheres were compared with monolayer cells, their responses to CT-179 were not identical. In all the models, the monolayer cells were more responsive to CT-179 treatment than the neurospheres. As summarized in Figure 2B, the lowest CT-179 dose needed to suppress cell proliferation >99% (IC99) was 0.03 µM in monolayer cells of ICb-1227 anaplastic astrocytoma (AA) and IC-3704GBM. The fact that these two models expressed vastly different levels of OLIG2 mRNA (>8-fold in ICb-1277AA, and <1-fold in IC-3704GBM) suggests that factors other than OLIG2 mRNA levels may have affected their responses to CT-179.

### 2.3. CT-179 Additively Enhanced XRT-Induced Cell Growth In Vitro

XRT is one of the mainstay therapies for children with high-grade gliomas. To examine whether inhibiting OLIG2 would promote XRT-induced cell death, we utilized high-throughput imaging technology to examine the change in cell number in FBS-containing media and neurosphere formation (similar to colony-forming efficiency) in serum-free media in cells from the same eight PDOX models (Figure 2B). Combining CT-179 (0.03~1 µM) with XRT (2~8 Gy) reduced the growth of monolayer cells and suppressed the formation of neurospheres, particularly in cells treated with lower CT-179 (0.03 and 0.1 µM) and XRT (2 Gy) doses (Figure 2C). Higher CT-179 (1 µM) and XRT (>4 Gy) doses as monotherapies were active, which partially explains the lack of additive effects for higher doses. These data provide encouraging results for the combination of CT-179 and XRT in pGBMs.

### 2.4. Oral Bioavailability and Effective Penetration of CT-179 Through the Blood–Brain Barrier (BBB)

**Figure 3:**
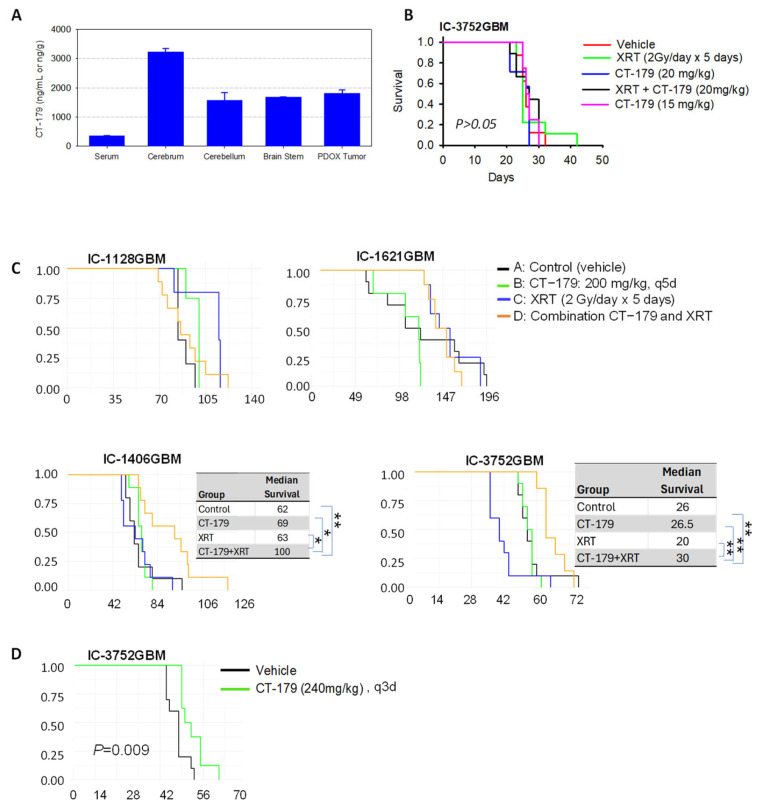
In vivo activities of CT-179 in PDOX models of pGBM treated with three different doses. (**A**) Determination of CT-179 concentration in vivo in normal mouse brain tissues and PDOX tumors following daily oral gavage of 24 mg/kg for 5 days compared with serum levels. (**B**) Log-rank analysis of animal survival times in IC-3752GBM treated with low (15–20 mg/kg) CT-179 alone and in combination with radiation (XRT). (**C**) Systematic analysis of animal survival times in four pGBM models treated with CT-179 (200 mg/kg) alone and in combination with fractionated XRT. The median survival times (days) are presented for the two models that responded to the combined treatment, together with the pair-wise comparison between the combination group and the remaining three groups. * *p* < 0.05, ** *p* < 0.01. (**D**) In vivo efficacy of an elevated CT-179 dose (240 mg/kg, gavage, every 3 days) in IC-3752GBM.

One of the barriers to brain tumor treatment is the difficulty of effective drug delivery through the BBB and/or blood–tumor barrier. Before we embarked on in vivo examination of therapeutic efficacy, we administered CT-179 (24 mg/kg) via oral gavage for 5 consecutive days to tumor-bearing mice and examined the oral bioavailability by determining drug concentration in normal brain tissues and PDOX tumors (Figure 3A). When compared with serum drug concentrations of 361.3 ± 1.5 ng/mL, oral gavage of CT-179 (24 mg/kg for 5 days) led to high-level drug penetration into murine cerebrum (3232.7 ± 569.2 ng/g), cerebellum (1563.3 ± 269.6 ng/g), and brain stem (1685.3 ± 309 ng/g). In the PDOX tumor tissues, the drug concentration reached 1814 ± 110.3 ng/g, slightly higher than that in the normal cerebellum and brain stem. These data showed that CT-179 penetrates the BBB, and the elevated CT-179 levels in normal brain tissues and PDOX tumors may have resulted from the accumulation of protein and/or brain binding of CT-179, although the fractions of free CT-179 need to be determined to better correlate with efficacy. Worthy of note is that we observed greater than 15% body weight loss in some of the treated mice. A reduced dose of 15 mg/kg was subsequently tested in non-tumor-bearing mice, in which no sign of toxicity was observed in all five mice.

### 2.5. Combining CT-179 and Fractionated XRT in PDOX Models Extends Survival Times

Radiation remains a major component of standard therapy for pGBM. Demonstrating an additive or synergistic anti-tumor activity by combining CT-179 with XRT would facilitate the initiation of clinical trials. To determine the therapeutic efficacy of CT-179 alone and in combination with XRT, we performed two sets of experiments. In the first set of experiments, we aimed to explore the toxicity and potential effects of combined low doses of CT-179 and radiation by treating one pGBM model (IC-3752GBM) at 15 mg/kg (oral gavage × 7 days) for the first week when combined with fractionated XRT that was delivered locally to the intra-cerebral tumor at 2 Gy/day for 5 days, followed by CT-179 as a single agent at 20 mg/kg doses 5 days/week in weeks 2–4. In all the animals, no signs of toxicity were observed. The animal survival times, however, were not significantly altered by CT-179 (both at 15 and 20 mg/kg) and/or fractionated XRT (Figure 3B), suggesting that the well-tolerated CT-179 at 15 mg/kg and 20 mg/kg failed to enhance the activities of XRT. Since OLIG2 is over-expressed in pGBMs, and its inhibition by CT-179 resulted in strong suppression of cell proliferation in vitro, we reasoned that the lack of in vivo activity was a result of ineffective drug delivery.

In the second set of in vivo experiments, we redesigned the treatment strategy by increasing the administration of CT-179 to 200 mg/kg every 4-5 days and expanded the number of pGBM PDOX models to four (Figure 3C). To treat pre-formed xenograft tumors, we implanted tumor cells (1 × 10^5^ cells/mouse) and allowed them to grow for 4–5 weeks before initiating treatment. CT-179 was administered via oral gavage at 200 mg/kg every 4 days (rapid-growing IC-3752GBM) or 5 days (IC-1128GBM, IC-1406GBM, and IC-1621GBM) for 8–10 total doses. XRT was delivered locally to the intra-cerebral tumor at 2 Gy/day for 5 days during week 2 of treatment. In all four models, CT-179 as a single agent did not significantly extend the survival times as compared with the untreated control groups, and significantly decreased survival times were observed in IC-1621GBM (*p* < 0.05) without major loss of body weight. Fractionated XRT acting alone was able to prolong the median animal survival times from 84 days to 121 days (*p* = 0.019) in one model (IC-1128GBM) but shortened survival times in IC-3752GBM and had no significant efficacy in the remaining two models.

Combining CT-179 with XRT significantly prolonged survival times in two models (IC-1406GBM and IC-3752GBM), as determined by pair-wise analysis using the Gehan–Wilcoxon method. In 1406GBM, the median animal survival times extended to 100 days in the combined treatment group, which was significantly longer than the 62 days (38% increase) in the control group (*p* = 0.0035), 66 days (34% increase) in the CT-179-only group (*p* = 0.0153), and 63 days (37% increase) in the radiation-only group (*p* < 0.0076) (Figure 3C) (Table 2), demonstrating synergistic anti-tumor activity between CT-179 and radiation. Similar results were obtained in IC-3752GBM, in which the median survival time of 30 days in the combined treatment group increased 15.6% compared with the control group (*p* = 0.0063), 13% compared with the CT-179-only group (*p* = 0.0006), and 50% compared with the XRT-only group (*p* = 0.0017) (Figure 3C) (Table 2), confirming the synergistic effects, although the overall levels of survival time extension remained modest.

To address the concern about potential off-target effects of the elevated drug concentration at 200 mg/kg every 5 days, we further increased the dose of CT-179 to 240 mg/kg every 3 days and treated IC-3752GBM (Figure 3D). Although differences in the survival times between the treated and the control groups reached statistical significance (*p* = 0.009), the actual extension of median survival time was <5%. When combined with the previously described survival data of 15 mg/kg, 20 mg/kg (Figure 3A), and 200 mg/kg (Figure 3C), these data showed dose-dependent efficacy of CT-179 in IC-3752GBM and suggested very limited off-target effects when treated with 200 mg/kg every 5 days. Comprehensive results from the in vivo testing experiments are provided in Table 2.

### 2.6. Protein Expression of OLIG2 in pGBM Cells Was Suppressed by CT-179

**Figure 4:**
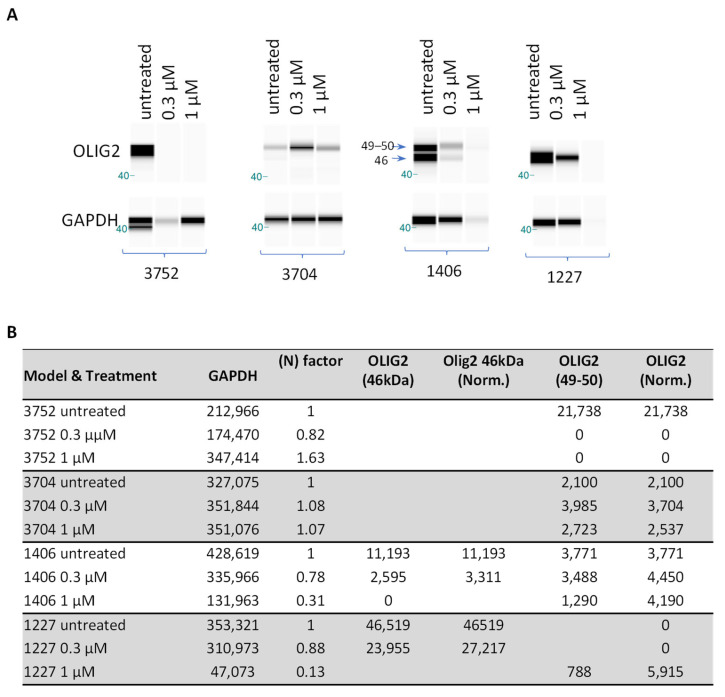
Changes in OLIG2 protein expression following in vitro treatment of CT-179. (**A**) Cells from 4 pGBM models were incubated with CT-179 (0.3–1 µM) for 3 days, and the changes in OLIG2 protein levels were examined through Western hybridization. GAPDH was included as a loading control. Two positive bands at 49–50 kDa and 46 kDa are indicated with arrows. (**B**) Summary of semi-quantitative analysis of OLIG2 levels.

Since CT-179 exhibited strong dose-dependent activities both in vitro and in vivo, we reasoned that suppressed OLIG2 protein levels may have played a role. To examine the impact of CT-179 doses on OLIG2 protein expression, primary cultured tumor cells from four PDOX GBM models were exposed to CT-179 (0.3 and 1 µM) for 3 days. In the untreated cells, Western hybridization identified OLIG2 at 49 kDa in all four lines, which agreed with the mRNA over-expression. Of note is that an additional band of OLIG2 at 46 kDa was detected, suggesting the complex protein expression of OLIG2 in pGBMs (Figure 4A). Additionally, we observed some inconsistencies in IC-3752GBM in which the GAPDH level in 0.3 µM-treated cells was much lower than the control and 1 µM-treated cells, while in IC-1406GBM and ICb-1227GBM, 1 µM treatment appeared to have caused significant cell killing, hence the low GAPDH levels. Semi-quantitative analysis of band intensity was therefore performed to compare the changes (Figure 4B). Decreased OLIG2 protein was observed in three out of four models, among which the complete suppression of OLIG2 protein was detected in IC-3752GBM, and significant reductions were observed in IC-1406GBM and ICb-1227AA (Figure 4). These data highlight the inter-tumoral heterogeneity of OLIG2 protein suppression by CT-179 in pGBM models and correlate with their in vitro responsiveness.

## 3. Discussion

The need for more effective and less toxic therapies is immense for pediatric high-grade gliomas. In this study, we showed that *OLIG2* is over-expressed in seven out of ten (70%) of the pediatric malignant gliomas analyzed and demonstrated that the elevated expression was well preserved in matching PDOX models even over serial in vivo sub-transplantations in mouse brains. Targeting *OLIG2* with the novel inhibitor CT-179 in vitro in matching pairs of monolayer and neurosphere cultures from eight PDOX-derived cells exhibited both time- and dose-dependent inhibition of cell proliferation. Furthermore, we showed that orally administered CT-179 can effectively pass through the BBB and accumulate in the normal cerebral, cerebellar, and brain stem tissues of SCID mice, as well as in intra-cerebral PDOX tumors, thereby overcoming one of the major barriers to systemic brain tumor treatment. In vivo therapeutic efficacy was examined in a stepwise approach with varying doses and frequencies aimed at maximizing efficacy and preventing excessive toxicity. While as a single agent, CT-179 was largely inactive in vivo, highlighting the malignant and therapy-resistant nature of pGBM, combination with XRT resulted in significant prolongation of survival in two out of four pGBM models in comparison to the control group. However, the addition of CT-179 to XRT significantly prolonged survival compared to XRT alone for only one pGBM model, for which the XRT-only group performed worse than the control group, and for another model, the addition of CT-179 to XRT significantly shortened survival compared to XRT alone. These data suggest that further pre-clinical approaches need to be explored to better support the targeting of OLIG2, to identify the biomarkers of response or resistance, and to optimize CT-179-containing combination regimens for pGBM.

Multiple lines of evidence have suggested an important role of *OLIG2* in driving pGBM growth. It is almost exclusively expressed in the CNS and codes for the development of oligodendrocytes, glia, and motor neurons from neuro-progenitors. In agreement with previous reports, we also observed *OLIG2* over-expression in pGBM and DIPG tumors; in a minority of tumors, *OLIG2* expression was greater than 10-fold higher than in the normal childhood brain tissues. Additionally, the high-level *OLIG2* expression was well maintained in 7/10 PDOX models. This finding not only supports the biological role of *OLIG2* in perpetuating tumor growth but also demonstrates that our PDOX model systems are clinically relevant and molecularly accurate for evaluation of the role of *OLIG2* as a novel therapeutic target for pGBMs.

CT-179 is a small-molecule inhibitor of *OLIG2*. It not only demonstrated time- and dose-dependent anti-tumor activities in vitro in cells from eight pGBM models but was also active in both monolayer cells (enriched with rapidly proliferating and “differentiated” tumor cells) and neurospheres (enriched with cancer stem cells), and monolayer cells appeared more responsive than neurosphere cells. Since a recent study on SHH medulloblastoma by Desai et al. provided strong evidence that CT-179 targets an OLIG2-driven transition from quiescence to proliferation in cancer stem cells of SHH-MB [28], our finding that monolayer cells were generally more responsive to CT-179 than neurospheres in pGBM presents an interesting contrast to a simple model in which CT-179 primarily targets stem cell activation. It is possible that in pGBM, OLIG2’s role in maintaining the proliferation of the bulk tumor cells (represented by monolayer cultures) is also a critical point of vulnerability, or pGBM stem cells might be less dependent on the specific OLIG2-mediated quiescent-to-active transition. A detailed experiment with OLIG2 knockdown in pGBM cells, both stem and non-stem tumor cells, in the near future should help to clarify the role of direct OLIG2 inhibition.

To achieve effective therapeutic efficacy in brain tumors, successful drug delivery through the BBB is frequently required. Our analysis of CT-179 concentrations in mice bearing pre-formed PDOX tumors provided a clinically relevant opportunity to evaluate the penetration of CT-179 into tumors and, at the same time, into normal brain tissues. The significantly elevated drug concentrations detected in the normal brains and PDOX tumors compared to serum concentrations was critical data that supported our subsequent large-scale in vivo efficacy studies in multiple PDOX models. To further understand the pharmacokinetics and pharmacodynamics of CT-179 in pGBMs, more animal models and extended time points are needed to determine the length of time (T_1/2_) that the drug remains in the brain and in tumors to optimize the dose and frequency for maximized therapeutic efficacy. One additionally encouraging aspect of CT-179 is that it exhibited a favorable safety profile in vivo in our animal models. All the mice tolerated the treatment very well and showed no significant loss of body weight during the therapies, whether administered alone or in combination. This finding is further supported by a recent study in which Li et al. demonstrated minimal off-target effects of CT-179 in 12 normal human primary cell lines at biologically relevant concentrations using the BioMAP^®^ Diversity PLUS^®^ platform, a cell-based screen used to assess the safety of pre-clinical experimental therapies on normal healthy tissues [27].

Single agents frequently show limited efficacy in cancer therapy. Despite multiple lines of evidence in the current study that suggested potential single-agent efficacy of CT-179 in pGBMs (strong in vitro activity and capability of passing through the BBB), it was largely not active as a single agent in vivo despite multiple adjustments of dose (from 15 to 240 mg/kg) and frequency (daily to every 3–4 days) in any of the four pGBM PDOX models tested. Although these PDOX models were highly malignant and difficult-to-treat tumors, including models derived from recurrent tumors (IC-1621GBM and IC-3752GBM), incomplete inhibition of *OLIG2* by CT-179 may have limited the overall efficacy. Indeed, analysis of *OLIG2* protein changes following CT-179 treatment in vitro suggested inter-tumoral heterogeneity and the need for relatively high doses (≥0.3 µM) for complete inhibition of *OLIG2* protein expression. The inclusion of more pGBM models treated with a broader range of CT-179 doses and time points should further elucidate the mechanisms and impact of inhibiting OLIG2 protein expression on overall therapy responses. Although medulloblastoma and pGBM are distinct biological entities, recent findings on CDK4 upregulation and the subsequent success of CDK4/6 inhibitors in overcoming CT-179 resistance [27] also provide a new therapeutic rationale for pGBM. Additionally, the potential reasons for CT-179 monotherapy failure in vivo suggest the possibility of rapid adaptive resistance, insufficient duration of target inhibition at effective concentrations, or tumor microenvironment-mediated protective effects, as well as decreased interference with OLIG2 dimerization or phosphorylation [27] in pGBMs.

Combination with XRT, an important component of standard care in pGBM, resulted in significant improvement of animal survival times in two out of four models, which is similar to our initial observation in vitro. Although the increased event-free survival times in the combination-treatment group were less than 1-fold (44% in IC-1406GBM and 15% in IC-3752GBM), the effects were statistically synergistic. Worthy of note is that we also observed a shorter animal survival time in the combination group than with XRT treatment alone in IC-1128GBM, suggesting that the potentiation of XRT toxicity by CT179, or vice versa, may exist in certain contexts. Additionally, it is necessary to understand the underlying mechanisms in the two models that failed to respond to the combined therapies. As shown in Table 1, all the models involved in the current study carry gene mutations that are distinct in the specific models. Although some common mutations, such as TP53, PIK3CA, and DNA mismatch repair deficiency genes (MSH6 and PMS2), were shared in several models, these mutations do not directly correlate with the in vivo responses. While this observation highlights intra-tumoral heterogeneity, it also suggests that responses to the OLIG2 inhibitor, despite mRNA over-expression, are relatively complicated, and more studies are needed. Further efforts still need to be made to explore the cellular and molecular mechanisms of the synergistic activities, including alteration of cell cycle distribution, putative cancer stem cells, and genetic/epigenetic alterations, to facilitate the development of combination therapies that are rationally and/or mechanistically designed and can be swiftly implemented or integrated into clinical trials. Indeed, DNA damage has long been recognized as one of the primary mechanisms of XRT-induced tumor cell killing. Our previous study showed that 2 Gy/day for 5 days alone caused massive (nearly 100%) expression of γH2AX in PDOX models of pediatric GBM and, at the same time, triggered the exit of putative cancer stem cells from the quiescent G_0_ phase into active proliferation [37]. Since OLIG2 expression is closely related to cancer stem cells, suppression of this critical gene by CT-179 may have contributed to the increased anti-tumor activity of XRT in the current study.

In summary, we utilized a relatively large panel of pGBM PDOX models to identify *OLIG2* as a therapeutic target in pediatric malignant gliomas and demonstrated that the novel *OLIG2* inhibitor CT-179 was active in suppressing pGBM cell proliferation both in monolayer and 3D neurosphere cultures (in a time- and dose-dependent manner) in eight pGBM PDOX models. Orally administered CT-179 was able to cross the BBB and accumulate in the brain and PDOX tumors, and importantly, significantly prolonged animal survival times in two out of four PDOX models when combined with fractionated XRT. Comparisons of combination-treated groups with CT-179 alone and XRT-only groups showed statistical evidence of a synergistic benefit from the addition of CT-179. These data provide key support for additional in vivo studies to optimize the dosing, frequency, and duration of CT-179 treatment to further improve its therapeutic efficacy, as well as a strong pre-clinical rationale for the initiation of clinical trials for patients with pGBM.

## 4. Materials and Methods

### 4.1. OLIG2 Inhibitor

CT-179 was supplied by Curtana Pharmaceuticals, Inc. (Austin, TX, USA). It is a potent and selective OLIG2 inhibitor with a molecular weight of 397.3. For in vivo use, a 30 mg/mL enteral suspension of CT-179 was prepared by mixing the drug powder with hydroxypropyl methylcellulose, tween 80, 0.1 N HCl, dimethylacetamide, and water and sonicating for 15 min.

### 4.2. PDOX Mouse Models

A total of 10 PDOX mouse models of pediatric high-grade glioma (pHGG), previously known as pediatric glioblastoma (pGBM) (n = 8), and diffuse intrinsic pontine glioma (DIPG) were included (Table 1). Cellular and molecular characterization have demonstrated their faithful replication of the histopathological features and molecular profiles of the original patient tumors [38,39,40,41]. Seven of these models were established from freshly resected brain tumor specimens collected from pediatric patients undergoing surgery at Texas Children’s Hospital in Houston, TX (Figure 1A), after informed consent was obtained from the patient or legal guardian in accordance with our local Institutional Review Board-approved protocol. Two DIPG models were established from autopsied tumor tissues. NOD/SCID mice were bred and housed in a specific pathogen-free animal facility at Texas Children’s Hospital. The inclusion of patient samples was based on availability; no patient sample was excluded. All animal experiments were conducted using an Institutional Animal Care and Use Committee (IACUC)-approved protocol, as described previously [36,38,42]. Tumor tissues were mechanically dissociated within 60 min of surgical removal. After the cell suspensions were passed through 40 and 100 μM cell strainers, viable tumor cells were dissociated into single cells, and small clumps (~5–10 cells) were counted with Trypan blue staining. Tumor cells (1 × 10^5^) were then suspended in 2 µL of culture medium and injected into mouse cerebra, 1 mm to the right of the midline, 1.5 mm anterior to the lambdoid suture, and 3 mm deep, via a 10 µL 26-gauge Hamilton Gastight 1701 syringe needle, as described previously [38,39,41]. The animals were monitored daily until they developed signs of neurological deficits or became moribund, at which time they were euthanized.

### 4.3. In Vitro Treatment with CT-179 Alone and in Combination with XRT

CT-179 was dissolved in dimethyl sulfoxide to generate a stock solution of 2 mM for in vitro experiments. To determine the time- and dose-dependent effects of CT-179 as a single agent, pGBM tumor cells were incubated as a monolayer in standard DMEM media supplemented with fetal bovine serum (FBS) and as 3D neurospheres in serum-free media composed of neurobasal media supplemented with EGF (50 ng/mL), bFGF (50 ng/mL), N2, and B27, as described previously. These cells were seeded into 96-well plates (2000–6000 cells/well) and exposed to CT-179 ranging from 0.03 to 10 µM or vehicle control for 14 days. Cell viability was measured on days 1, 4, 7, 10, and 13–14 using the Cell Counting Kit-8 (CCK-8) (Dojindo Molecular Technologies, Rockville, MD, USA) [38,42]. To examine the effects of CT-179 in combination with XRT, matching monolayer and neurosphere cells were exposed to CT-179 (0.03–1 µM) and/or XRT (2–8 Gy). Cell numbers were quantified via high-throughput imaging technology.

### 4.4. In Vivo Analysis of CT-179 Concentrations in the Brains of SCID Mice

To test the capability of CT-179 to pass through the blood–brain barrier, mice were treated with CT-179 (24 mg/kg/day by mouth) once daily for 5 days. These mice were euthanized 1 h after the last treatment, and their plasma, brain tissues (cerebrum, cerebella, and brain stem), as well as orthotopic xenograft tumors, were harvested for analysis of drug concentrations. The body weight of the treated mice was measured daily.

### 4.5. In Vivo Treatment of PDOX Mouse Models with CT-179 and Standard Therapy

Cryopreserved xenograft tumor cells were retrieved, quickly thawed, washed, and immediately injected into the brains of SCID mice (1 × 10^5^ cells in 2 µL of medium), as described previously [38,42,43]. The implanted tumor cells were allowed to grow for 2–4 weeks to form solid tumors approximately 1–2 mm in size by 2 weeks and 3–5 mm by 4 weeks, as shown in our previous analysis of serial sections of whole mouse brains [38].

For each pGBM model, 40 mice were implanted with PDOX cells within 2 h and randomly divided into 4 groups (n = 10 per group) by cages (5 mice/cage): control, CT-179, XRT, and CT-179 + XRT. Sample size was estimated with SigmaPlot 14. All treatments were started 1–5 weeks after intra-cerebral (IC) tumor cell implantation, depending on the tumor growth rate, i.e., treatment of fast-growing tumors (IC-3752GBM) was started 1 week post-tumor implantation, while treatment of slower-growing tumors was postponed to weeks 4–5 (IC-1406GBM, IC-1621GBM, and IC-1128GBM) to allow for the formation of equivalently sized solid xenograft tumors (1–3 mm in size). Bias reduction was achieved by having at least two investigators double-check the dose and drug administration. For CT-179 treatment, mice were administered CT-179 (200 mg/kg) via oral gavage every 5 days. XRT was administered using an X-ray irradiator (RS-2000) (Rad Source Technology, Buford, GA, USA) and was delivered locally onto the right cerebral xenograft tumor via a 3 mm thick custom-made lead shield with a 10 mm hole. XRT was dosed at 2 Gray/day once daily for 5 days during the second week of treatment, as detailed previously [44,45]. Animal body weight was monitored weekly as a surrogate marker of toxicity. To determine survival benefits from these treatments, the mice were monitored daily until they developed neurological deficits or became moribund, at which time they were euthanized and their brains removed for analysis.

### 4.6. Capillary Western Blot Analysis

Protein quantification was performed using the Wes automated capillary Western system (Bio-Techne, Minneapolis, MN, USA, formerly ProteinSimple, #004-600) with a 66–440 kDa Separation Module (SM-W006). All procedures and buffers were from the manufacturer. Samples were diluted to 25 μg/mL in sample buffer (100-fold diluted 10× Sample Buffer 2 from the Separation Module), mixed with Fluorescent Master Mix, and denatured at 95 °C for 5 min. Approximately 40 nL of sample was automatically loaded per capillary, resulting in 1–10 ng of total protein analyzed per reaction. Samples, antibody diluent (anti-Rabbit Detection Module DM-001), primary antibodies, HRP-conjugated secondary antibodies, and chemiluminescent substrate (luminol/peroxide) were pipetted into a pre-filled assay plate according to the manufacturer’s instructions. Primary OLIG2 rabbit antibodies (#65915, Cell Signaling Technology, Danvers, MA, USA) were diluted at 1:500 in antibody diluent. GAPDH rabbit antibodies (#14C10, Cell Signaling Technology) were diluted 1:50 in antibody diluent. Secondary anti-rabbit antibodies conjugated to HRP (#7074, Cell Signaling Technology) were diluted at 1:1000 in antibody diluent. The assay was run using default instrument settings: stacking and separation at 475 V for 30 min, blocking for 5 min, primary and secondary antibody incubations for 30 min each, and chemiluminescence detection for approximately 15 min with exposures of 1, 2, 4, 8, 16, 32, 64, 128, and 512 s.

Electropherograms were analyzed using Compass software (version 5.0.1, Bio-Techne), and protein peaks were quantified by calculating the area under the curve (AUC). Automatic peak detection was manually verified, and peaks with signal-to-noise ratios ≥ 10 and peak height-to-baseline ratios ≥ 3 were considered positive. A 6-point calibration curve of a reference control sample (ranging from 0.004–0.250 μg of total protein) was included in each run, requiring linearity R^2^ > 0.99 and a chemiluminescence signal > 150,000 arbitrary units at the highest concentration point. Target protein expression was normalized to GAPDH as a loading control. Final protein levels were calculated as a percentage of control (%CTRL) using the formula: %CTRL = (sample protein equivalent to × μg/mL control from calibration curve ÷ x μg/mL sample loaded) × 100, followed by normalization to GAPDH %CTRL.

### 4.7. Statistical Analysis

Values were presented as mean ± standard deviation (SD). The effect of treatment on cell proliferation was analyzed with two-way analysis of variance (ANOVA). Changes in animal survival times were analyzed using the Kaplan–Meier method, followed by pair-wise comparisons using the Gehan–Wilcoxon test. No new code was developed. All statistical analyses were performed using SigmaPlot 14.0 (Systat Software, Inc., San Jose, CA, USA). *p* < 0.05 was considered statistically significant.

## 5. Conclusions

This study was designed to address a clinical need for new therapies for pediatric high-grade glioma (pHGG), previously known as glioblastoma (GBM). Using a total of 10 PDOX models, we confirmed the over-expression of *OLIG2* in pHGG patient tumors as well as in matching PDOX models, thereby identifying OLIG2 as a new molecular target. We subsequently revealed strong in vitro anti-tumor activities of CT-179, a newly developed OLIG2 inhibitor, in both monolayer and 3D neurosphere cultures, when used alone and in combination with radiation, and demonstrated its unique capacity to pass through the blood–brain barrier. In vivo therapeutic efficacy of orally administered CT-179 showed that CT-179 monotherapy was active at high doses and that combining it with clinically relevant fractionated radiation (2 Gy/day × 5 days) led to significant extension of animal survival times in two out of four PDOX models. These data provide preclinical evidence to support the targeting of OLIG2 with CT-179 in pHGG patients.

## Figures and Tables

**Table 1:** List of PDOX models of pediatric brain tumors.

No.	Model ID	Age/Sex	Diagnosis	Recurrent	Mutation
1	IC-1621GBM *	6 y	GBM	Yes	MSH6 (R1263H), TP53
2	IC-1406GBM	5/F	GBM	No	PMS2 (S46I), TP53
3	ICb-1227AA	16.9/F	GBM (radiation induced)	Yes	PAX8, EIF5B, FOXd2
4	IC-1128GBM	8.7/M	GBM	Yes	PIK3CA, TP73, TP53, RB1, NF1
5	IC-3752GBM	5/F	GBM	Yes	COL4A4, EEFSEC, SLC27A5,
6	IC-2305GBM	9/M	GBM (small cell variant)	No	TP53, H3F3A (G35R), ATRX
7	IC-3704GBM	12/M	GBM (small cell variant)	No	SUFU, PR3R1
8	IC-1502GBM	4.7/M	GBM (small cell variant)	No	NF1, BRAF (R726G)
9	IBs-W0128DIPG	8.4/M	DIPG	Yes	PI3CA, UBE4A, CBX4
10	IBs-P1215DIPG	7/M	DIPG	Yes	NOTCH3, SOX11, FGF13

Note: * IC = Intracerebral; ICb = Intra-cerebellar, IBs = Intra-brain stem; AA = Anaplastic astrocytoma; GBM = glioblastoma; DIPG=diffuse intrinsic pontine glioma.

**Table 2:** Summary of statistical analysis of animal survival times following the treatments.

Model and Treatment Groups	Summary of Survival							Between Groups	Pair-Wise Comparison			
	N	Nd	Nx	Na	Nev	KM med	KM 95% LCL		EFS T-C	EFS T/C	Net Log Cell Kill	*p*-Value Gehan-Wilcoxon
***IC1128GBM***												
**A.** Vehicle (6.67 μL/g q5 days)	5	0	0	5	5	84	84					
**B.** CT-179 only (200 mg/kg q5 days)	5	0	1	4	4	100	90	**A** vs. **B**	16	1.19	0.11	0.0292
**C.** XRT (2 Gy × 5 days)	5	0	0	5	5	115	115	**A** vs. **C**	31	1.37	0.22	0.0976
**D.** Combination (CT-179 +XRT)	10	0	1	9	9	86	76	**A** vs. **D**	2	1.02	0.01	0.9474
								**B** vs. **C**	15	1.15	0.09	0.1213
								**B** vs. **D**	−14	0.86	−0.08	0.2407
								**C** vs. **D**	−29	0.75	−0.15	0.1051
***IC1406GBM***												
**A.** Vehicle (6.67 μL/g q5 days)	10	0	0	10	10	62	58					
**B.** CT-179 only (200 mg/kg q5 days)	10	0	1	9	9	69	66	**A** vs. **B**	7	1.11	0.07	0.1243
**C.** XRT (2 Gy × 5 days)	9	0	0	9	9	63	52	**A** vs. **C**	1	1.02	0.01	0.6212
**D.** Combination (CT-179 +XRT)	10	0	1	9	9	100	72	**A** vs. **D**	38	1.61	0.37	0.0035
								**B** vs. **C**	−6	0.91	−0.05	0.3954
								**B** vs. **D**	31	1.45	0.27	0.0153
								**C** vs. **D**	37	1.59	0.35	0.0076
***IC-1621GBM***												
**A.** Vehicle (6.67 μL/g q5 days)	10	0	0	10	10	113.5	85					
**B.** CT-179 only (200 mg/kg q5 days)	10	0	5	5	5	120	105	**A** vs. **B**	6.5	1.06	0.03	0.6685
**C.** XRT (2 Gy × 5 days)	10	0	2	8	8	149	133	**A** vs. **C**	35.5	1.31	0.19	0.2875
**D.** Combination (CT-179 +XRT)	9	0	1	8	8	145	138	**A** vs. **D**	31.5	1.28	0.17	0.3521
								**B** vs. **C**	29	1.24	0.15	0.0003
								**B** vs. **D**	25	1.21	0.13	0.0003
								**C** vs. **D**	−4	0.97	−0.02	0.6384
***IC-3752GBM***												
**A.** Vehicle (6.67 µL/g q5 days)	10	0	0	10	10	26	25					
**B.** CT-179 only (200 mg/kg q5 days)	10	0	0	10	10	26.5	25	**A** vs. **B**	0.5	1.02	0.01	0.7584
**C.** XRT (2 Gy × 5 days)	10	0	0	10	10	20	18	**A** vs. **C**	−6	0.77	−0.14	0.0008
**D.** Combination (CT-179 +XRT)	10	0	3	7	7	30	30	**A** vs. **D**	4	1.15	0.09	0.0063
								**B** vs. **C**	−6.5	0.75	−0.15	0.001
								**B** vs. **D**	3.5	1.13	0.08	0.0006
								**C** vs. **D**	10	1.5	0.3	0.0017

Note: N = total number of mice entering experiment, Nx = number of additional mice excluded from analysis, Na = number of mice in analysis, Nev = number of events, KM med = Kaplan-Meier estimate of median time-to-event (days), EFS T − C = difference in median time-to-event (days) between Treatment and Control groups.

## Data Availability

The original contributions presented in this study are included in the article. Further inquiries can be directed to the corresponding author.

## Abbreviations

The following abbreviations are used in this manuscript:

pHGG	Pediatric high-grade glioma
GBM	Glioblastoma
PDOX	Patient-derived orthotopic xenograft
OLIG2	Oligodendrocyte lineage transcription factor 2
XRT	Radiation
DIPG	Diffuse intrinsic pontine gliomas
